# Vascular Contributions to Cognitive Impairment and Treatments with Traditional Chinese Medicine

**DOI:** 10.1155/2016/9627258

**Published:** 2016-11-23

**Authors:** Xinhua Zhou, Guozhen Cui, Hisa Hui Ling Tseng, Simon Ming-Yuen Lee, George Pak Heng Leung, Shun Wan Chan, Yiu Wa Kwan, Maggie Pui Man Hoi

**Affiliations:** ^1^State Key Laboratory of Quality Research in Chinese Medicine and Institute of Chinese Medical Sciences, University of Macau, Macau; ^2^Department of Bioengineering, Zunyi Medical University, Zhuhai Campus, Guangdong, Zhuhai, China; ^3^State Key Laboratory of Chinese Medicine and Molecular Pharmacology, Department of Applied Biology and Chemical Technology, The Hong Kong Polytechnic University, Hong Kong; ^4^Department of Food and Health Sciences, Faculty of Science and Technology, Technological and Higher Education Institute of Hong Kong, Hong Kong; ^5^School of Biomedical Sciences, The Chinese University of Hong Kong, Hong Kong

## Abstract

The prevalence of cognitive impairment and dementia caused by cerebrovascular disease is likely to increase with the global aging population. Vascular contributions to cognitive impairment and dementia (VCID) is a wide spectrum term used to include a diverse heterogeneous group of cognitive syndromes with vascular factors regardless of the cause of pathogenesis. VCID ranges from mild cognitive impairment to full-blown dementia with vascular dementia (VaD) as the most severe stage. It is further complexed by the coexistence of other forms of dementia such as Alzheimer's disease (AD). Recent researches in the functions of the neurovascular unit (NVU) suggest that dysfunction of the NVU might be the cause of primary vascular events in the brain that leads to further neurodegeneration. In this review, we have briefly summarized various forms of VCID. There is currently no standard therapy for VCID or dementia. Given the fact that Traditional Chinese Medicine (TCM) has gained popularity worldwide, we also reviewed recent scientific and clinical findings on various antidementia TCM for the treatment of VCID, including* Salvia miltiorrhiza, Huperzia serrata, Ligusticum chuanxiong, Ginkgo biloba, Panax ginseng,* and also TCM formula Sailuotong capsule (SLT) and Fufangdanshen tablets (FFDS).

## 1. Introduction

Dementia, which describes a syndrome with a gradual decline in cognitive functioning, is a spectrum term that includes various forms of cognitive impairment especially among the elderly of our society. In 2015, the World Health Organization (WHO) estimated 47.5 million people are living with dementia worldwide, and the number is projected to be tripled to 135.5 million by 2050 [1]. The overall prevalence of dementia among people aged 60 years and above is between 5 and 10%, varying among different global regions [2, 3]. In 2010 in China, 9.19 million of people were living with various forms of dementia, and the prevalence increases quickly with age escalating from 2.6% at age 65–69 to 60.5% at age 95–99 [4]. The most common type of dementia is Alzheimer's disease (AD), which accounts for 50–70% of all cases registered, followed by VaD, which accounts for 25% [1, 2]. Age-related dementia is a major cause of disabilities in the elderly. Apart from the financial burdens, the social stigma associated with the loss of cognitive abilities and dependency on others also causes psychological distress in patients as well as their families. The epidemic scale of dementia poses one of the biggest challenges on global public health systems and the financial burden associated with the social care needed. The pathogenesis of dementia is complex and it involves the interactions between many different physiological systems. Traditionally, AD and VaD are classified clinically as neuropathological and cerebrovascular disorders. However, patients with AD often have mixed etiologies with both neurodegenerative and cerebrovascular pathologies [3, 5, 6]. Besides, ischemic or hemorrhagic cerebrovascular diseases or cerebral lesions caused by cardiovascular origin are commonly associated with cognitive impairments [3, 5]. Cerebral infarctions and alterations in brain blood vessels commonly occurred in the elderly which are possibly due to age-related degeneration and other diseases [7, 8]. Since the brain is a highly perfused organ and requires a continuous blood supply for its physiological functions, it is not surprising that damage to the cerebral circulation are associated with an increased risk of many types of dementia. In addition, epidemiology evidence indicates that AD and VaD share similar cardiovascular risk factors including apolipoprotein E (APOE*ε*4), hypertension, hypercholesterolemia, obesity, and diabetes [5, 9, 10], although the strength of the association between AD and cardiovascular risk factors could be greatly influenced by the designation of parameters, and further evaluation is needed [11, 12]. Therefore, recent researches in neurodegenerative disorders such as dementia and AD have focused on understanding the interplay between vascular dysfunctions and primary neurodegenerative processes. It is also suggested that cerebrovascular pathologies may be primary causes as well as contributing factors in the progression of cognitive impairment and dementia.

In this review, we have included an overview of the definitions of various forms of dementia with vascular origins, the importance of the neurovascular unit (NVU), and the preclinical and clinical investigations of using Traditional Chinese Medicine (TCM) to treat and manage cognitive decline and dementia. Management of vascular risks and symptoms is the primary approaches in treating vascular dementia, and TCM has proven ability to treat cardiovascular diseases and hypertension effectively [3, 13]. Moreover, TCM formulations for the treatment of dementia-like and memory disorders have been extensively documented in the classical Chinese medical literature, including herbs such as* Salvia miltiorrhiza*,* Huperzia serrata, Ligusticum chuanxiong, Ginkgo biloba,* and* Panax ginseng* [14–16]. In a recent meta-analysis study, it indicated that TCM exhibited comparable efficacy and safety as Western medicine for improving the cognitive and behavior functions of patients with vascular cognitive impairment with no dementia [17]. Therefore, it is proposed that TCM has great potential uses as preventive strategies against dementia which can have positive impacts on global public health.

## 2. Definitions of Vascular Dementia (VaD), Vascular Cognitive Impairment (VCI), and Alzheimer's Disease (AD)

Dementia describes a group of syndromes relating to cognitive decline. Clinical manifestation of different forms of dementia exhibits different levels of impaired performance in various cognitive domains, including memory, learning, executive function, and behavioral changes (e.g., depressive symptoms). Cognitive impairment ranges from mild to severe declines in any cognitive domain. Alzheimer's disease (AD) is the most common form of dementia, followed by VaD [1, 2]. In the literature, “VaD” is often used ambiguously to describe a group of clinically similar cerebrovascular disorders associated with multiple pathological features, such as multi-infarcts, single infarcts, hemorrhages, white matter hyperintensities. Moreover, the progression of the manifestation of dementia is preceded by a prodromal stage in which the patient experiences progressive cognitive decline but is still able to maintain independent daily activities [5]. This has further led to the usage of the general terms, for example, VCI or VCID, in order to include all cognitive impairments caused by cerebrovascular abnormalities, which could be quite confusing. Essentially, VCI or VCID is used to include a diverse heterogeneous group of cognitive syndromes with vascular origins regardless of the cause of pathogenesis, ranging from mild cognitive impairment to full-blown dementia, and VaD is the most severe stage [3, 8, 18]. The heterogeneity and complexity of VCI however make it difficult for appropriate clinical characterization. Currently there are no accepted pathological criteria for clinical diagnosis for VCI, but identifiable subtypes of VCI have been categorized. This classification system may be useful for clinicians to diagnose for the prevention and the treatment of cognitive dysfunctions. The characterization and classification of VCI are beyond the scope of the present article, and more detailed information could be found in previous reviews [3, 19, 20]. In addition, AD and VaD have traditionally been characterized as separate disorders based on the clinical diagnostic criteria. However, the “pure” form of AD or VaD is not commonly found; instead most people with dementia have mixed pathologies. Besides, AD frequently coexists with cerebrovascular abnormalities such as alterations in vascular structures (e.g., cerebral amyloid angiopathy, CAA) and cerebral infarctions. Accumulating evidence suggested that AD and VaD have additive effects and probably interact with each other. It has been suggested that cerebrovascular dysfunction could play a role in the development and progression of AD. Therefore, the vascular component in the mixed pathology is particularly important for the understanding of the pathogenies of dementia. In the next section, we will give a very brief overview of the major subtypes of VCI. Note that the categorization of VCI described here does not mean to replace any existing clinical criteria for the characterization of different types of VaD but to offer a simplified overview for a better understanding of various VCI terminologies, especially for nonclinician researchers. The terms VCI and VCID are used interchangeable in this article.

### 2.1. Major Subtypes of Vascular Contributions to Cognitive Impairment (VCI)

Many subtypes of VCI or VCID have been described before. The disorder is sometimes classified according to the location of vascular lesions, the causative vascular mechanisms, and their clinical manifestations. Chronic cerebral ischemia and arteriosclerosis were originally thought to be the cause of vascular contributed dementia but later discovered that it is cerebral infarcts rather than ischemia that causes dementia [21]. Therefore, we try to summarize the major subtypes of VaD according to the type of infarcts exhibited. Similar clinical features of VaD subtypes have been described under different names; therefore we also try to include them into specific groups. In brief, the major subtypes of VCI include vascular mild cognitive impairment (VaMCI), multi-infarct dementia (MID), strategic-infarct dementia (SID) (which is also commonly known as subcortical vascular dementia (SVD) or small vessel disease (SVD)), poststroke dementia, and mixed dementia of AD and VaD. SID (or SVD) accounts for over 40% of all VCI reported [20, 21]. Recent studies also indicated that mixed dementia particularly VaD in conjunction with AD is also commonly observed among elderly people [20, 21].

#### 2.1.1. Vascular Mild Cognitive Impairment (VaMCI)

Dementia is preceded by a stage in which mild cognitive decline is first manifested in individuals without affecting care-free daily independent activities. VaMCI is used to describe this intermediate stage between normal cognition and dementia caused by vascular diseases with the presence of cognitive impairment but it is not severe enough to fit into the criteria for VaD [22]. An equivalent stage occurring in AD is referred to as mild cognitive impairment (MCI) and is used to identify people who are at risk for the later amnestic stage of AD. The prevalence of VaMCI is twice as much of VaD [22]. VaMCI is potentially treatable by management of vascular risk factors and diseases [3, 22]. Moreover, VaMCI is usually associated with other diseases such as heart failure, autoimmune disorders, and depression, and previous studies showed that cognitive functions of patients could be improved with respective treatments for these disorders with or without specific treatment for VaMCI [22–24]. VaMCI is common in stroke patients shortly after attacks, and in some patients cognition may improve as part of the stroke recovery process [25, 26].

#### 2.1.2. Large-Vessels VaD: Multi-Infarct Dementia (MID)

MID is caused by the “synergistic effects” caused by multiple vascular lesions (“multiple mini strokes”) in the brain irrespective of specific location or volume. The mini strokes which disrupted the blood flow to the brain may occur without noticeable clinical symptoms, and over time these lead to irreversible injuries in the brain tissues. MID can be diagnosed by brain imaging techniques such as computed tomography (CT) and magnetic resonance imaging (MRI) scan. MID usually affects people between the ages of 60–75 and is more common in men than in women. However, MID is not the single most common type of VaD in elderly people; instead patients are more likely to have mixed dementia with both AD and VaD pathology (discussed later) [27].

#### 2.1.3. Small-Vessels VaD: Subcortical Ischemic Vascular Dementia (SIVD)

SIVD is caused by the occlusion of small penetrating arteries which supply blood to the inner structures of the brain which is recognized as Binswanger's disease, lacunar infarct (LACI), or small vessel disease (SVD). SIVD is also known as silent brain infarction (SBI), and it is the most common type of VaD with 20–40% incidence in our community [28, 29]. This disease can be diagnosed using brain imaging methods, for example, CT and MRI scan. Hypertension is the major risk factor for SVID, and it is therefore potentially preventable and treatable [29]. The hereditary genetic vasculopathy Cerebral Autosomal-Dominant Arteriopathy with Subcortical Infarcts and Leukoencephalopathy (CADASIL), which causes 11% of lacunar stroke cases with leukoaraiosis in middle-aged adults, is one of the best examples of small vessel disease affecting mainly the small penetrating cerebral and leptomeningeal arteries [30].

#### 2.1.4. Strategic-Infarct Dementia (SID)

It has been traditionally recognized that single or few focal infarcts occurred in some functionally important brain regions (“strategic”) such as lesions in the thalamus, caudate nucleus, lenticular nucleus, angular gyrus, and internal capsule. However, the concept of strategic infarction is under reexamination with larger prospective MRI studies to study the relationship between the extent and location of lesions and the cognitive networks [3].

#### 2.1.5. Poststroke Dementia (PSD)

Poststroke dementia (PSD), or poststroke cognitive impairment includes any type of dementia that occurs after stroke, irrespective of the leading causes, which can be vascular, neurodegenerative, or mixed. Having a stroke doubles the risk of dementia development. The prevalence of PSD in patients is about 30%, which varies between age, races, diagnostic criteria, and periods after stroke [31]. The underlying causes of cognitive impairment after stroke are not known at present. It is suggested that vascular lesions caused by ischemia/hypoxia or hemorrhages, cerebral microbleeds, white matter lesions, and neurodegenerative pathologies from other conditions such as AD all contribute to and probably interact with each other to the pathogenesis of PSD. Increasing age is a major determinant of PSD development. It has been reported that 15% of stroke survivors at the age of 60–69 had new-onset dementia, and the prevalence was greatly increased to 26% at the ages 70 to 79 and 36% at the age over 80 years [31–34].

#### 2.1.6. Mixed Dementia

Mixed dementia is a condition in which abnormal features of more than one type of dementia occur simultaneously in the brain, and over 44% of dementia was in fact mixed dementia with a combination of AD and VaD pathologies [35]. The coexistence of AD and vascular lesions is particularly common in older patients. Clinically, a spectrum of vascular diseases is related to the failure of microvasculature functions to regulate cerebral circulation and elimination of interstitial fluid and solutes in both AD and VaD. In AD, abnormal accumulation of amyloid-beta (A*β*) in the brain arterial walls, a condition known as cerebral amyloid angiopathy (CAA), leads to the weakening of the brain blood vessel wall and increases the risk of hemorrhages [36]. More recently, dementia research has focused on the importance of the neurovascular unit (NVU) at the level of cellular and molecular mechanisms (discussed later) [6, 30, 37]. Mixed dementia may also include cases of AD and VaD associated with any other disorders such as Parkinson's disease (PD) and dementia with Lewy bodies (DLB) [35].

The following shows general classification for cognitive impairment with vascular components.

Pathological features includes white matter hyperintensities (WMHs), multiple or single infarcts, hemorrhages, alteration of brain vessel structure, and cerebral hypoperfusion.

*Vascular Mild Cognitive Impairment (VaMCI)*

- Predementia stage: patients have cognitive decline without affecting daily independent functioning
- Symptoms: amnestic, lack of attention, expressive language disorder, and visual depth perception
- Diagnosis methods: brain imaging, cerebrospinal fluid tests, and so forth

The following shows severe cognitive decline.

*Vascular Dementia (VaD)*

Large-vessel VaD
*Multi-infarct dementia (MID)*
    - Caused by multiple infarcts (cortical and/or subcortical) with synergistic effect irrespective of location or volume
    - Symptoms: getting lost, language problem, apathy, performing difficulties, emotional lability, and loss of social skills
    - Diagnosis methods: a neurological exam, a history of stepwise mental decline, computed tomography (CT) or magnetic resonance imaging (MRI) scans, an electroencephalogram, a transcranial Doppler, and so forth
Small vessel VaD
*Subcortical ischemic vascular dementia (SIVD)* (may also be known as* Binswanger's disease, Lacunar infarct (LACI), silent brain infarction (SBI), small vessel disease (SVD) (e.g., CADASIL))*
    - Caused by occlusion of penetrating arteries and thus reduction of blood supply to the brain's deep structures
    - Symptoms: sudden hemiparesis, pseudobulbar palsy, small-stepped gait, urinary incontinence, dysarthria, dementia, and changes in effect including inappropriate laughing or crying
    - Diagnostic methods: assessment of cognitive deficits (criteria of clinical trial of SIVD), CT or MRI, and so forth
Strategic-infarct dementia (SID)
  - Caused by focal infarcts in functionally important brain regions (concept of SID is under reexamination)
  - Symptoms: mental deterioration, depression, emotional lability, apathy, cognitive, and other deficits
  - Diagnostic methods: MRI, CT, perfusion SPECT, and so forth

*Poststroke Dementia (PSD)*

- Including any type of dementia that occurs after stroke irrespective of causes
- Symptoms: trouble with speaking and understanding, trouble with seeing in one or both eyes, cognitive impairment
- Diagnostic methods: CT, PET, functional MRI, spectroscopy, and so forth

*Mixed Dementia*

- Coexistence of more than one type of dementia (e.g., VaD, Alzheimer's disease (AD), and Parkinson's disease (PD))
- Symptoms: symptoms may vary, depending on the type of disease, it may be similar to those of AD or another specific type of dementia
- Diagnostic methods: CT, MRI, and so forth

## 3. Cerebral Circulation, the Neurovascular Unit (NVU), and VCI

The pathogenesis underlying VCI has remained elusive with many complexities. However, vascular risk factors associated with the cerebral circulation such as hypertension and stroke are shared by many neurodegenerative diseases and are associated with the development of dementia [5]. The cerebral circulation is responsible for one of the most important jobs in the body to provide and regulate blood supply for the highly energy demanding central nervous system. Given the highly perfused nature of the brain, it is not surprising that any disruption or dysfunction in the cerebral circulation will lead to cognitive impairments. The brain is perfused by a sophisticated network of cerebral blood vessels. The anatomy and cellular organization of the cerebral vasculature have been described in detail previously [38, 39] and it is briefly introduced here.

The common carotid arteries carry blood from the heart to the brain and branch into two pairs of carotid arteries (the internal and external carotid arteries). The carotid arteries then give rise to cerebral arteries which further divide into pial arteries that run along the surface of the cortex within the pia-arachnoid. As the pial arteries penetrate into the parenchyma, they become progressively smaller, becoming intracerebral arterioles and cerebral capillaries to supply blood to the corresponding regions of the cerebral cortex. Importantly, the structures and functions of the vessels change significantly as they penetrate into the brain's inner structure. All cerebral vessels have a layer of highly specialized endothelial cells (the blood-brain barrier, BBB) that provide specific barrier functions to regulate the fluid and solute exchange between the brain and the blood. The pial arteries on the surface of cerebral cortex consist of a layer of endothelial cells surrounded by 2-3 layers of smooth muscle cells and an outer layer of perivascular nerves. The smooth muscle layers are important for supporting the endothelium and regulating vessels contractility, similar to the systemic blood vessels. As the vessels penetrate in the brain and become smaller intracerebral arterioles, the smooth muscle layer becomes thinner and the vessels are completely wrapped around by astrocytic end-feet and other glia cells. The glia (including astrocytes, oligodendrocytes, and microglia) become more and more important in the maintenance and stabilities of the endothelium. As the vessels further progress and become cerebral capillaries, their endothelial cells are sealed by tight junctions, enveloped by pericytes (which replace the smooth muscle layer), and then surrounded by a continuous basement membrane (BM). End-feet of perivascular astrocytes cover the brain capillaries BM and act to integrate the communication between the endothelium and neurons.

**Figure 1:**
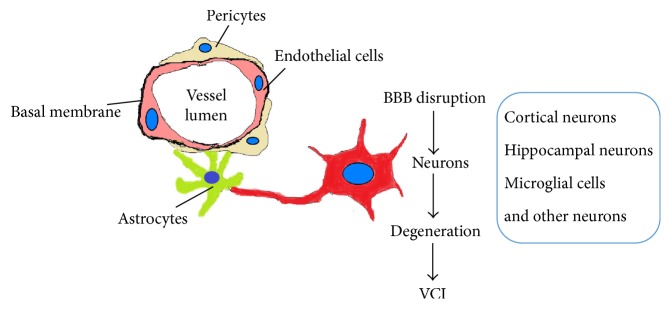
Schematic illustration of the cellular structure of the neurovascular unit (NVU). Dysfunction of the NVU causes blood-brain barrier (BBB) disruption and leads to the degeneration of neurons and VCI.

The integrated system within a cerebral capillary that is responsible for microvascular homeostasis and neurovascular coupling (the correlation between local neuronal activity and changes in blood flow or hyperemia) is now considered a specialized functional unit known as the neurovascular unit (NVU) (Figure 1). The current understandings of the multicellular organization and functions of the NVU in normal and disease conditions are well described in recent reviews [6, 30, 39]. Essentially, the components of the NVU include all the cells present within the brain capillary (brain endothelial cells, astrocytes, pericytes, microglia, neurons, and circulating inflammatory cells) as well as the basal membrane and endothelial glycocalyx (a network of membrane-bound proteoglycans and glycoproteins covering the luminal-side of the endothelium). It is proposed that vascular risk factors such as hypertension and hypercholesterolemia damage the NVU and lead to chronic hypoperfusion, hypoxia, inflammatory activation, and oxidative stress. The formation of a hypoxic microenvironment at the NVU also directly contributes to inflammatory activation by regulating gene such as hypoxia-inducible factor 1*α* (HIF1*α*), which serves as inducer of other genes involved in inflammatory response signaling. Tissue hypoxia and inflammatory activation in neurodegenerative diseases induce the NVU to undergo various cellular interactions and adaptations in responses, exhibiting pathophysiological features such as blood flow perturbation, vascular alterations, adaptive angiogenesis, vascular remodeling, BBB permeabilization, loss of tight junction and/or adherens junction proteins, endothelial injuries, pericytes retraction, BM breakdown, neurovascular uncoupling, and neuronal impairments. This highlights the association of the injury at NVU and the disruption of cerebral microcirculation as a crucial step in ischemic-hypoxic tissue damage leading to neuronal degeneration and cognitive impairment. It is suggested that dysfunction of the NVU may participate in all brain pathologies development, and the primary microvascular events may be etiological to neurological diseases, as well as being involved in diseases manifestation. Epidemiological studies have also provided strong evidence supporting that the NVU dysfunction is closely related to some neurological pathogenesis. For example, hypertension in animal models causing brain vessel rarefaction and a reduction in capillary density and microvessel formation [40]. In the hereditary CADASIL disease of small cerebral arteries, the NVU has been indicated as a predominant causative factor in the pathogenic mechanism of the disease. Structural alterations in the small penetrating cerebral and leptomeningeal arteries in CADASIL caused by pathogenic mutations in NOTCH3 (Notch homolog 3) lead to NVU impairment, causing cerebral blood flow reduction and subcortical ischemia, with lacunar infarcts correlating with cognitive decline [30]. Recently, the NVU has also been proposed to play an important part in the development of cognitive decline observed in AD [30, 37, 41]. It is reported that dementia is more likely to happen when vascular lesions coexist in AD patients. Apart from cerebral amyloid angiopathy (CAA) (the accumulation of amyloid-beta (A*β*) in brain blood vessels), an increased concentration of soluble A*β* peptides in the cerebral circulation of AD patients is also observed [42]. The elevated level of soluble A*β* causes oxidative stress, and the damage on cerebral circulation induced by A*β* precedes cognitive decline, thus suggesting that cerebrovascular dysfunction might be involved in the pathogenesis of cognitive impairment [42]. All these observations emphasize the importance of the interaction between vascular and neuronal systems in the context of disease pathogenesis and imply the potential of the NVU as a target for disease prevention, treatment, and management.

## 4. Therapeutic Implications for VCI with Traditional Chinese Medicine (TCM)

Currently there is no standardized treatment regimen for VCI. Several pharmacological agents approved for AD (donepezil, galantamine, rivastigmine, and memantine, Table 1) have been tested to treat patients with VaD with only modest improvements on standard cognitive measures and failed to achieve regulatory approvals [3]. Other antidementia candidates for treating cerebral small vessel disease are also developed (e.g., nimodipine, huperzine) [3]. Uncertainties in diagnostic criteria and the measurements of cognitive domains further pose challenges and hurdles to the progress of drug research and development. Given the fact about the long history of TCM development and uses, many Chinese herbs have been identified for the potential treatment of the dementia-like disorders [43]. VCI belongs to the category of memory disorders according to TCM theories that is known as jian wang (*健忘*) (“forgetfulness”) and dai bing (呆病) (“dementia”) [16, 17]. Over the last decades, many clinical trials were conducted in China to investigate TCM for treatment of cognitive impairment and dementia. Despite the enormous efforts involved, most of these trials conducted were not well-designed with limitations such as small sample size, the lack of being truly randomized, double-blind, and placebo-controlled, and the incomplete cognitive assessments dedicated for VaD [44]. Thus, the genuine effectiveness of most TCM therapies assessed for treating dementia remains to be verified. Nevertheless, these studies provided a valuable resource for the search of potential antidementia drugs and therapies [16, 17, 45]. In addition, the most effective approach for VCI treatment is the management of underlying vascular risk factors and prevention of further cerebrovascular injury. Many TCM herbal ingredients have proven abilities to prevent and treat cardiovascular diseases and management of hypertension and stroke. In the following session, the experimental studies of the herbs frequently reported for the treatment of memory disorders with vascular origins are reviewed and discussed (Table 1).

### 4.1. *Salvia miltiorrhiza*

The dried root of* Salvia miltiorrhiza*, also known as Danshen, is a widely used TCM for the treatment and prevention of cardiovascular diseases (CVDs) such as angina pectoris, myocardial infarction, and arteriosclerosis. According to TCM theory, Danshen promotes blood flow and resolves blood stasis. Several registered pharmaceutical products containing Danshen extracts such as Danshen Dripping Pill and Fufang Danshen Tablet are widely used in clinics in the China showing effectiveness for treating CVDs [43]. The herbal extract of Danshen contains water-soluble danshensu (DSS) and salvianolic acid B (Sal B) and lipid-soluble tanshinone I (Tan I), tanshinone IIA (Tan IIA), cryptotanshinone, and dihydrotanshinone. DSS is the major component in Danshen which possesses multiple pharmacological effects that are beneficial to the cardiovascular system, including coronary artery dilatation, antiarrhythmia, microcirculation improvement, protection of myocardial ischemia/reperfusion injury, and antiplatelet aggregation [46–48]. Danshen has been used for the prevention and treatment of cerebral infarction and the underlying mechanisms involved multiple pathways, including antihypotension, antiplatelet aggregation, and anti-inflammatory and antioxidative effects [49]. Recent animal in vivo studies have also shown that DSS exhibited protection against cerebral ischemic/reperfusion injury [50] and neuronal cytotoxicity against oxidative damage [51]. Diabetes is a vascular risk factor shared by AD and VaD, and the spatial learning and memory are impaired in diabetic animals. In cognitive impaired model of streptozotocin-induced diabetic mice, DSS administration ameliorated the learning and memory deficits by attenuating advanced glycation end products- (AGE-) mediated neuroinflammation [52]. The lipid-soluble Tan IIA improved learning and memory deficits in a chronic cerebral ischemia rat model in vivo by protection against free radical insults and regulating the levels of glutamate and *γ*-aminobutyric acid (GABA) [53]. On the other hand, Sal B exerted various neuroprotective and anti-inflammatory activities in vivo and in vitro [54].

### 4.2. *Huperzia serrata*

An alkaloid isolated from the Chinese herb* Huperzia serrata*, Huperzine A, is a natural cholinesterase inhibitor that selectively inhibits acetylcholinesterase activity and increases acetylcholine levels in brain, thereby improving cognitive functions in patients with dementia [55]. In fact, Huperzine A has been used for treating AD and mild memory deficits since 1994 in the China [55, 56]. In a randomized, double-blinded placebo-controlled clinical trial with the participation of 78 patients who have mild to moderate VaD, Huperzine A treatment significantly improved the cognitive functions in these patients [57]. Furthermore, both animal and human safety evaluations have demonstrated that Huperzine A is safe [58]. Comparing with other acetyl cholinesterase inhibitors such as galantamine, donepezil, and tacrine, Huperzine A showed a longer duration of action, better penetration of the blood-brain barrier, higher oral bioavailability, and fewer adverse reactions [59].

### 4.3. *Ligusticum chuanxiong*

**Figure 2:**
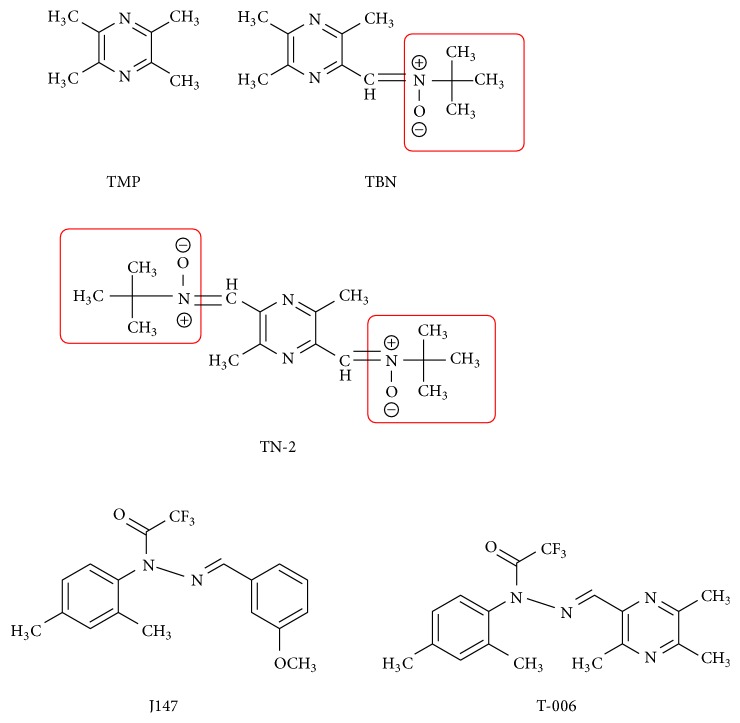
The structures of derivatives of TMP.

*Ligusticum chuanxiong* is a famous medicinal herb known for its therapeutic effects for treating ischemic stroke and tetramethylpyrazine (TMP) is the major active ingredient. TMP possesses many protective cardiovascular effects, including protection of mitochondrial functions, inhibition of lipid peroxidation by free radicals, attenuating calcium (Ca^2+^) overload, maintaining calcium homeostasis, inhibiting inflammatory reaction, improving endothelial cell function, and inhibiting myocardial cell apoptosis [60]. Besides, TMP inhibits angiogenesis [61], platelet aggregation, and antithrombosis and ameliorates microcirculation [47, 62]. An injectable formulation known as Guanxinning Injection (or Danshen Chuanxiong Injection) which contains extracts of Danshen and Chuanxiong in combination has displayed a high efficacy in treating hypertension and other cardiovascular diseases [63]. Apart from the beneficial cardiovascular effects, TMP is a potent neuroprotective agent. Evidence from in vivo experiments in rats with middle cerebral artery occlusion (MCAO) demonstrated that TMP has potent neuroprotective effects against ischemic brain injuries caused by transient focal cerebral ischemia reperfusion [64–67]. It has been shown that TMP effectively reduced the size of cerebral infarction and brain edema, suppressed oxidative stress and inflammatory and apoptotic responses [64, 66, 67], and could salvage neurological functions, neuronal dendritic plasticity, and behavioral disturbance in the MCAO stroke model in rats [65, 67]. In another study with scopolamine-induced memory impairment model in rat, TMP could also effectively reverse memory deficits and preserved postsynaptic protein synthesis by restoring the impaired cAMP/PKA/CREB pathways [68]. Besides, TMP attenuated iron-induced oxidative damage and apoptosis in rat cerebellar granule cells [69]. Derivatives of TMP, known as TBN and TN-2, which was synthesized with the addition of powerful free radical-scavenging nitrone moiety (Figure 2), exhibited promising neuroprotective effects. TBN exhibited stronger antioxidative properties without affecting the thrombolytic activity of the parent TMP and produced neuroprotection against ischemic brain injuries in rat transient and permanent MCAO stroke models via the prevention of Ca^2+^-mediated cellular damage caused by Ca^2+^ influx and overload [70, 71]. Furthermore, TBN protected and rescued dopaminergic neurons from 1-methyl-4-phenylpyridinium- (MPP^+^-) and methyl-4-phenyl-1,2,3,6-tetrahydropyridine- (MPTP-) or 6-hydroxyldopamine- (6-OHDA-) induced damage in vitro and in vivo models of Parkinson's disease (PD) by the reduction of oxidative stress plus increased cellular antioxidative defense capacities [72]. TN-2, which has two nitrone moieties, also exhibited potent neuroprotective effects against 6-OHDA-induced neurotoxicity and MPTP/MPP^+^-induced dopaminergic neurons damage [73, 74]. Another derivative of TMP, known as T-006 (Figure 2), protected primary cortical neurons from neurotoxicity as well as improvement in memory deficits in APP/PS1 transgenic mice [75].

### 4.4. *Ginkgo biloba*

*Ginkgo biloba* is a widely cultivated tree since ancient China with the sacred belief for its health-promoting properties. The fruits and leaves of Ginkgo biloba are used to treat various types of diseases and symptoms such as atherosclerosis, diabetes, poor circulation, fatigue, vertigo, tinnitus, and cognitive disorder [15]. A standardized extract of Ginkgo biloba (EGb 761) has been shown to exhibit potent antioxidative and antiplatelet effects and could effectively reduce damage from cerebral ischemia [76–81]. Besides, EGb 761 extract protected hippocampal neuronal and neuroglia cells against damage and enhanced the recovery of learning/memory impairments from cerebral ischemia/reperfusion in mice and rats [79–83]. Furthermore, the Ginkgo extract has been reported to improve memory deficits and cognitive dysfunctions in various experimental models of memory disorders such as chronic stress, aging, and Parkinson's disease (PD) [84–86]. Recently, EGb 761 has also been shown to enhance the functions and integrity of cerebral microvascular endothelial cells under chronic stress induced by hypoxia, hyperglycemia, or amyloid-beta (A*β*) protein [87, 88], suggesting that functions of the neurovascular unit might play an important role in the protective effects of EGb 761. Interestingly, various clinical trials have reported the efficacy of EGb 761 in improving the cognitive impairment of patients with AD and VaD [89–92]. In a recent study of a 24-week randomized controlled trial [92], once-daily preparation of EGb 761 was shown to improve cognitive functions, neuropsychiatric symptoms, and functional abilities in 333 patients with AD and 71 with VaD. In the market, Ginkgo extract is developed into an herbal supplement called Gingium® which is for improving mild to moderate age-related cognitive impairments and alleviating symptoms of AD, VaD, and/or mixed dementia. It is noted that despite the encouraging evidence of the beneficial effects of Ginkgo biloba consumption in treating VaD, the potential drug-drug interactions especially associated with long-term usage of Ginkgo extract required further evaluation.

### 4.5. *Panax ginseng*

The root of the Panax ginseng is well-known for many health benefits and has been widely used to promote physical strength and healthy minds. Ginseng and its active pharmacological ingredients ginsenosides have been shown to improve age-related memory and cognitive deficits; their therapeutic effects for the treatments of cerebrovascular diseases and neurodegenerative diseases have been reviewed recently [93–95]. Several studies reported that total ginseng, total saponins, and Rg1, Rb1, and Rg2 have neuroprotective effects [96–99]. Rg1 promoted the proliferation of hippocampal progenitor cells after transient global ischemia experimental brain ischemia [99], whereas Rb1 has been reported to protect hippocampal neurons against ischemia [100]. In a recent study using a VaD rat model, Rg2 improved neurological performance and memory ability of VaD rats after cerebral ischemia reperfusion through the modulation of antiapoptotic signaling pathways [98]. Results from the experimental AD models have also demonstrated that ginseng extract and ginsenosides are effective in protecting and alleviating neuronal damage [93]. Two clinical studies on ginseng therapy for the treatment of AD reported a significant improvement in cognitive performance [101, 102].

### 4.6. TCM Formulae for Treatment of VaD

In recent years, various TCM formulae for the treatment of VaD have been developed, for example, Sailuotong capsule (SLT) and Fufangdanshen tablets (FFDS) [103, 104]. SLT is composed of ginseng extract (ginseng total saponins), Ginkgo biloba extract (Yinxingtong ester), and saffron extract (saffron total glycosides). The therapeutic functions of SLT are yi-qi-huo-xue and hua-yu-tong-luo according to the TCM theory. Pharmacodynamics studies showed that SLT could significantly improve neurological symptoms caused by focal cerebral ischemia and improve learning and memory ability in animal models of VaD. In healthy adults, one-week consumption of SLT improved both neurocognitive and cardiovascular functions [103]. More recently, an international multicenter phase II clinical trial of SLT in patients with mild to moderate VaD has been initiated in 2012 to 2014, and the clinical results/outcomes will be available soon [105]. In recent phase III clinical trial launched in 2016, which involved research teams of Xiyuan Hospital of the China Academy of Chinese Medical Sciences and the National Institute of Complementary Medicine at Western Sydney University, over 200 Australians with dementia were recruited and participated in this clinical trials, and results obtained from the pilot studies have implicated the effectiveness of SLT uses in improving learning and memory [106]. On the other hand, an ongoing double-blind, randomized, parallel placebo-controlled clinical study on the evaluation of efficacy and safety of FFDS (*Radix Salvia miltiorrhiza* formula tablets) for patients with mild to moderate VaD has shown encouraging data on treating cognitive symptoms in VaD patients [104].

## 5. Conclusions

VCI (or VCID) is a complex form of dementia, involving both vascular and neurological aspects. The heterogeneity of VCI and the coexistence of other forms of dementia such as AD make it even harder for effective drug development and clinical treatment. Recently, the important roles of the neurovascular unit (NVU) in neurodegenerative diseases are rigorously studied. Unfortunately, there is no standard therapy for VCI. Most Western medicines are target-oriented whereas TCM offers therapeutic outcomes via holistic approaches which are probably appropriate for treating VCI which involved various factors. Evidence-based TCM therapies have shown some promising results for treating the symptoms of cognitive impairments. Therefore, TCM serves as a rich resource for therapeutic developments for treating dementia, and it will be of interest to further investigate the actions of potential TCM ingredients on the functions of NVU.

## Figures and Tables

**Table 1:** Current therapeutic agents and TCM for the treatment against VCI/dementia and their action targets/mechanisms.

Therapeutic agents	Targets/mechanisms for VCI
Donepezil Galantamine Rivastigmine	Acetylcholinesterase (AChE) inhibition
Memantine	Noncompetitive NMDA receptor antagonist
*Salvia miltiorrhiza*	Antiplatelet aggregation, anti-inflammation, and antioxidative effects
*Huperzia serrata*	Selective AChE inhibition
*Ligusticum chuanxiong*	Antiapoptosis, antioxidant, anti-inflammation, antiplatelet, and block calcium (Ca^2+^) overload
*Ginkgo biloba*	Restoring mitochondrial dysfunction, improving neuronal energy supplement, improving compromised hippocampal neurogenesis and neuroplasticity, inhibiting A*β* protein aggregation, decreasing blood viscosity, and enhancing microperfusion
*Panax ginseng*	Antioxidant, antiplatelet, antihyperlipidemic, stimulation of NO production, improvement in blood circulation, and enhancement of vasomotor tone
Saffron	Antioxidant and inhibiting serotonin reuptake in synapses
